# On the Physiological Inferences to Be Deduced from the Structure of the Nervous System in the Invertebrated Classes

**Published:** 1839-10

**Authors:** 


### Art. XIV.

*On the Physiological Inferences to be deduced from the Structure of the Nervous System in the Invertebrated Classes of Animals.* By William B. Carpenter, m.d., m.r.c.s., &c.—*Edinburgh*, 1839. 8vo, pp. 83. (*With Two Plates.*)

We are induced to bestow an early notice upon this essay, because we have reason to know that it contains the results of enquiries upon which its author has been for some time engaged, and the publication of which has been delayed by his wish to embody them in the inaugural dissertation required at the time of graduation. To those who have looked with any degree of interest at the recent discussions upon the functions of the nervous system, we recommend the perusal of this pamphlet, as containing a summary of important facts, which have not before been thus compared and examined, and a series of inferences from them which have an immediate and, to our minds, decisive bearing upon the chief questions at issue. To our other readers it may be adduced as an example of the important aid which comparative anatomy is capable of affording to those who aim at penetrating the mysteries of physiological science; since an intricate question appears here to be easily decided by an appeal to the structure of the inferior classes of animals, which it would be difficult, and perhaps impossible to determine upon any evidence furnished by the study of man alone. We have only further to add, with regard to its general merits, that this essay has been honoured by one of the four prize-medals now annually given by the University of Edinburgh. This fact sufficiently stamps its value as a *thesis*, but is not enough to characterize it as it deserves. The readers of this Journal will understand the reserve with which we speak on this subject; and will not, we hope, misconstrue the delicacy which restrains us from giving more than a dry analysis of a production which, however prized by physiologists as an important addition to science, will, we think, be regarded by them yet more as a further earnest of what physiologists may expect from one who, almost in his pupilage, has rivalled, if not outstripped, the most learned of his contemporaries.

As many of our readers, however, may desire to know the general results of our author's enquiry, and the nature of the evidence upon which they are founded, without following him through the details of his argument, we shall present them with such a brief analysis of this essay as may serve the purpose. The opinion which it appears to be his object to establish is, that the centres of simple reflex action, and the nervous filaments which serve as its channels, are generally among the invertebrata structurally distinct from those which minister to sensation and voluntary motion. Some traces of such a view, in reference to the articulated classes, at least, may be found in our former pages (Vol. V. p. 500-1); but these could only be regarded as speculative, until confirmed or disproved by more extended enquiry. It has been by directing his attention to the anatomy of the nervous system in the mollusca, and by comparing it with the structure of the same apparatus in the articulata, that the author has succeeded in giving a high degree of probability to his doctrine, if not in fully establishing it. Those cases are among the most interesting in the history of science, in which a theory, erected upon the comparison of a limited number of instances, is found to be not only *applicable* to an entirely new group of phenomena, but to meet with *independent* support from them: and it is almost superfluous to observe upon the vastly-increased probability of its truth when such is the case.

The first two sections, which treat of the supposed nervous system of plants, and of the nervous system of the radiata, present no peculiar novelty, in fact or opinion, to the readers of this journal. We shall therefore pass on at once to the consideration of the leading characters of the nervous system of the mollusca. The chief of these is the smallness of the number of the ganglionic centres, and the distinctness in the function of each. Except in the highest group, the cephalopoda, in which there is a considerable tendency to approach the vertebrata, we rarely find more than four pairs of ganglia, and very frequently but three, or even two. Those which commonly form pairs often coalesce into a single one; and to such an extent is this reduction carried in some instances, that in one large group, the tunicata, we have but a single ganglion, and this is obviously connected chiefly with the respiratory apparatus. We must not suppose that there is here a *concentration* of function, such as we perceive in the central masses of vertebrata; the fact being, on the contrary, that the functions themselves are absent; so that, as the author has observed, nature has here anticipated the experiment of the physiologist, in showing that life may be continued with the integrity of that portion only of the nervous system which is concerned in the respiratory function. It is in the gasteropoda, the typical mollusca, that the peculiarities of the nervous system of the group may be best studied. Here we usually find one pair of ganglia situated above the œsophagus, and connected with the organs of special sensation. These, the *cephalic* ganglia, evidently present the nearest approach to the brain of vertebrata. The situation of the others is more variable. We usually find a single ganglion, or a pair, in connexion with the branchiæ; another with the foot; and sometimes another with the mantle. The distribution of their nerves to the different organs would alone indicate their respective functions; but these are placed beyond doubt by that very great variety in the disposition of these organs which is characteristic of the mollusca. The development of the sensory organs, the situation of the gills, the structure and position of the foot, and the conformation of the mantle, are well known to differ in the most obvious manner, in genera which are most closely allied to each other. Hence we are able, by the discovery of corresponding changes in the arrangement of the nervous system, to satisfy ourselves of the peculiar functions of its different centres.

These points being established, we are led to enquire into the influence of these centres upon one another, and we then perceive the important fact, that, while they have little or no communication with each other, they are all directly connected with the cephalic ganglia, which seem thus to harmonize and control their individual actions. Frequently, however, a communication seems to exist where there is really none,—a cord from one ganglion passing through another in its way to the head; but in such a case, we find a distinct communicating cord belonging to this second ganglion; and there are even instances in which three such cords are found, two of which have proceeded from ganglia of different functions *fused* into one mass, while the third has passed through this in its way from a distant centre. These facts have been previously noticed by anatomists; but no explanation of them has been given. Further, a careful examination of these ganglia and their connecting cords discloses this important fact, which is peculiarly evident in the case of the *pedal* ganglia,—that the cord does not lose itself in the gray matter of the ganglion, but divides itself into filaments which mix with those proceeding from it, to form the nervous trunks which it distributes. We can scarcely, then, fail to infer that the pedal ganglion, with the nervous fibrils proceeding from it, is the source of the reflex actions of this organ, whilst the filaments, which are continuous with those of the connecting trunk, and which thus proceed from the gray matter of the cephalic ganglia, are the channels of sensory impressions, and of the motor impulses of volition. Those who desire to become acquainted with the detailed evidence upon which this novel position is based must consult the essay; we have here only space for a curious example of its application. The arms of the cuttle-fish are provided, as is well known, with a series of suckers, which are important instruments of locomotion and prehension to this animal. It has been observed by Dr. Sharpey that the nerves which supply these arms are provided with ganglionic enlargements, of which one corresponds with each sucker; and that, like the ventral cord of the articulata, they are composed of two tracts, of which one passes continuously over the ganglia without entering them, but sends off nervous filaments, which help to form the branch going to each sucker. It has been supposed that the white tract is the motor portion; and the ganglionic has been thought to be the sensory; but Dr. Carpenter has shown that we may with much more probability regard the white tract as conveying the influence of the will to all the suckers alike, whilst the ganglion in connexion with each ministers to those reflex actions which it will exhibit even when separated from the rest of the arm.

The idea that there is any analogy between the ganglia of the sympathetic nerve, or those on the posterior roots of the spinal nerve (which the recent investigations of Remak have shown to be part of the sympathetic system,) and the ganglionic centres of the invertebrata, is, we think, very satisfactorily combated in this essay. It is plainly shown that the latter are analogous to certain portions of the cerebro-spinal system, both in their structure, connexions, and functions. Any inferences founded upon this fallacious analogy must therefore be invalid; and such we cannot but deem that which has attributed sensory functions to the ganlionic portion of the ventral cord of the articulata, and exclusively motor functions to its white or fibrous tract. This cord is evidently formed by *a repetition of the pedal ganglia* of the mollusca; and every pair of nerves given off from it is manifestly derived in part from its own ganglion, and in part from the cephalic ganglia, just as in that class. An easy explanation is afforded by this hypothesis, of the independence of the segments so remarkable in the articulata, and of the number and variety of the reflex movements which they will perform when isolated; and its claims to reception, as physiological truth, are evidently increased to no trifling amount by its conformity, not only with the analogy of the mollusca, as already stated, but with that supplied by vertebrata, which we shall give in the author's words:

"In the vertebrata we find the ganglionic or mixed portion of the spinal cord, and the simply fibrous tracts, performing functions respectively analogous; for, when any segment is isolated from the rest, reflex actions may be excited through it, in the production of which the white columns can scarcely participate, being structurally distinct from each other and from the ganglionic portion of the cord, and continuous only with the fibrous portion of the brain; whilst pathology supplies us with instances of the converse occurrence, namely, the destruction of the ganglionic portion by disease, without the functions of the parts below being impaired—*their* ganglionic portion being segmentally independent, and their communication with the brain being maintained by a continuity of white or fibrous structure." (pp. 61-2.)

We have not here space to follow our author through another very interesting branch of his enquiry,—that which relates to the *stomatogastric* system of nerves in the invertebrata. Here also may be seen the advantage of not studying one class of animals to the exclusion of another; for here again the functions of certain problematical nerves in insects receive important elucidation by comparison of them with analogous parts in the mollusca. The general result of this comparison is a very interesting one,—that in the lower tribes, the system of nerves concerned in manducation and deglutition is as distinct from that which may be designated as the sensori-volitional system as is that of respiration; whilst in vertebrata, these different centres are united, and placed in more immediate relation to each other—the centre of the stomato-gastric system, as well as that of the respiratory, being in the medulla oblongata.

The following is the recapitulation furnished by our author of the chief results which he considers to have been established or rendered probable by the facts which he has adduced. We quote them without further remark at the present time, as meriting our general assent, although several points in them lie open to discussion.

"A general review of the ground over which we have passed will enable us, we think, to draw the following conclusions with a high degree of probability:

"1. That a nervous system, in the form of connected filaments with ganglia on certain parts of them, exists in all animals, (that is, in all beings endowed with any degree of sensibility and voluntary power,) although its presence may not be detected by our means of observation.

"2. That the actions most universally performed by a nervous system are those connected with the introduction of food into the digestive cavity.

"3. That we have reason to regard this class of actions as everywhere independent of volition, and perhaps also of sensation;—the propulsion of food along the œsophagus in man being of this character.

"4. That, for the performance of any action of this nature, a nervous circle is requisite, consisting of an *afferent* nerve on the peripheral extremities of which an impression is made; a ganglionic centre, where the white fibres of which that nerve consists terminate in gray matter, and those of the efferent nerve originate in like manner; and an *efferent* trunk conducting to the contractile structure the motor impulse, which originates in some change in the relation between the gray and white matter.

5. That such actions may be regarded as the simplest of those which the nervous system performs, and most resemble the examples of contraction produced by the irritation of distant organs in plants (where an *impression* is mechanically conveyed by the circulating system,) of any which the animal kingdom affords.

"6. That in the lowest animals such actions constitute nearly the entire function of the nervous system; the amount of those involving sensation and volition being very small.

"7. That, as we ascend the scale, the evidence of the participation of true sensation in the actions necessary for the acquirement of food, as shown by the development of special sensory organs, is much greater; but that the movements *immediately* concerned with the introduction of food into the stomach remain under the control of a separate system of nerves and ganglia, to the action of which the influence of the cephalic ganglia,—the *special*, if not the *only*, seat of sensibility and volition,—is not essential.

"8. That, in like manner, the active movements of respiration are controlled by a separate system of nerves and ganglia, and are not dependent upon that of sensation and volition, though capable of being influenced by it.

"9. That the centres of these systems are brought into closer structural relation with that of the sensori-volitional system as we ascend the scale of invertebrated animals; until they at last apparently become a part of it, as in Vertebrata, where, however, they still remain really separate, and may be artificially insulated.

"10. That, whilst the actions of these systems are in the lower tribes almost entirely of a simply reflex character, we find them, as we ascend, gradually becoming subordinated to the will; and that this is effected by the mixture of fibres proceeding directly from the cephalic ganglia with those arising from their own centres.

"11. That the locomotive organs, in like manner, have their own centres of reflex action, which are independent of the influence of volition, perhaps also of sensation.

"12. That the influence of the will is conveyed to them by separate nervous fibres, proceeding from the cephalic ganglia; and that similar fibres probably convey to the cephalic ganglia the impressions destined to produce sensations.

"13. That the stomato-gastric, respiratory, and locomotive centres are all united in the spinal cord of vertebrata, where they form one continuous ganglionic mass; and that the nerves connected with all these also receive fibres derived immediately from the cephalic ganglia.

"14. That whenever peculiar consentaneousness of action is required between different organs, their ganglionic centres are united more or less closely; and that the trunks themselves are generally connected by bands of communication.

"15. That the sympathetic system does not exist in the lowest classes in a distinct form; that the nervous system of the invertebrata, taken as a whole, bears no analogy with it; that, as the divisions of this become more specialized, some appearance of a separate sympathetic presents itself—but that this is never so distinct as in vertebrata.

"16. Hence it may be inferred that, as the sympathetic system is *not* developed in proportion to the predominant activity of the functions of organic life, but in proportion to the development of the higher division of the nervous system, its office is not to "preside over" the former, but to bring them into relation with the latter; so that the actions of the organs of vegetative life are not dependent upon it, but influenced by it in accordance with the operations of the system of animal life." (pp. 76-9.)

We must not conclude this notice without a few words in reference to Dr. Marshall Hall and his opinions. It will be recollected that, on a former occasion, we withheld our assent from that portion of his doctrines which concerned the *structural distinctness* of his *excito-motor* from the *sensori-volitional* system. We by no means asserted that it might not prove to be well founded, but simply that no sufficient evidence was adduced by him in its behalf. On the latter point we see no reason to change our opinion. A new field of enquiry has been explored by one who was originally adverse to this part of Dr. Hall's system; and he has obtained evidence of its truth, which ought to be gratifying to that gentleman, and which will not, we hope, pass unnoticed by him. "A wise man," it has been well said, "changes his mind as often as he sees occasion for it." We do not pretend to infallibility; and we see no discredit in giving our assent to opinions which we formerly hesitated in receiving, when new evidence is presented in their support. As to Dr. Hall's *entire originality* in this part of his doctrines, we never expressed the slightest doubt; and we are now quite willing to accord to him the credit of having made a most important advance in nervous physiology, not only (as we formerly stated) by "the harmonious combination" of previously-entertained doctrines "into a uniform system, and the application of it to the explanation of many phenomena which were not formerly regarded as explicable on such principles," but by the discovery of a previously-unsuspected fact, which, when placed entirely beyond doubt, will take the rank of those established by Sir C. Bell.

